# Trace Element Concentrations in Drinking Water and Urine among Saharawi Women and Young Children

**DOI:** 10.3390/toxics6030040

**Published:** 2018-07-21

**Authors:** Inger Aakre, Sigrun Henjum, Elin Lovise Folven Gjengedal, Camilla Risa Haugstad, Marie Vollset, Khalil Moubarak, Tecber Saleh Ahmed, Jan Alexander, Marian Kjellevold, Marianne Molin

**Affiliations:** 1 Institute of Marine Research, 5817 Bergen, Norway; 2 Department of Nursing and Health Promotion, Faculty of Health Sciences, OsloMet-Oslo Metropolitan University, 0310 Oslo, Norway; Sigrun.henjum@oslomet.no (S.H.); marian.kjellevold@hi.no (M.K.); mmolin@oslomet.no (M.M.); 3 Faculty of Environmental Sciences and Natural Resource Management, Norwegian University of Life Sciences, 1433 Aas, Norway; elin.gjengedal@nmbu.no (E.L.F.G.); camilla.haugstad@gmail.com (C.R.H.); marie.vollset86@gmail.com (M.V.); 4 Saharawi Ministry of Public Health, 37000 Rabouni, Algeria; fadala2001@gmail.com (K.M.); tecberas@gmail.com (T.S.A.); 5 Division of Infection Control and Environmental Health, Norwegian Institute of Public Health, 0403 Oslo, Norway; jan.alexander@fhi.no; 6 Bjorknes University College, 0456 Oslo, Norway

**Keywords:** drinking water, urine, trace elements, chemical elements, thyroid dysfunction

## Abstract

Poor water quality has been reported along with a variety of negative health outcomes in the Saharawi refugee camps in Algeria. We assessed the concentration of elements in drinking water and urine in refugee women and children. Twenty-four samples of distributed public drinking water were collected, along with urine samples from 77 women and 296 children. Using inductively coupled plasma mass spectrometry, we analyzed water and urine for 31 and 10 elements, respectively. In addition, the water samples were analyzed for five anions by ion-exchange chromatography. Data were described according to two areas: zone 1 with purified water and water with naturally better quality, and zone 2 with only partially purified water. Most elements in drinking water had significantly higher concentration in zone 2 compared with zone 1. Sodium, chloride, nitrite, and nitrate were the parameters that exceeded the WHO Guidelines for Drinking Water Quality. Among both women and children, urinary concentration of vanadium, arsenic, selenium, lead, iodine, and uranium exceeded reference values, and most of the elements were significantly higher in zone 2 compared to zone 1. Even though water purification in the Saharawi refugee camps has increased during the last years, some elements are still exceeding the WHO guidelines for drinking water quality. Moreover, urinary exposure of some elements exceeded reference values from the literature. Further effort should be made to improve the water quality among the Saharawi refugees.

## 1. Introduction

Although essential for life, water can also be a source of harmful substances. Food, water, and air are the major sources of exposure to potential toxic chemical substances, of which water and food are considered the most important sources when work-related exposure is unaccounted [1]. The sustainable development goals (SDG 6A) draws attention to the importance of supply of clean and safe drinking water [2]. Access to clean drinking water is a human right [3], and The World Health Organization (WHO) states that safe drinking water is defined as water that does not represent any significant risk to health over a lifetime of consumption [4]. Moreover, the WHO has established guideline values for the upper acceptable level of elements and anions in drinking water [4].

Different biomarkers, e.g., urine, serum, or hair, may be used to assess the level of exposure to elements [5,6]. Urinary excretion and serum concentrations may be good indicators for assessing on-going exposure or body burden [7]. Biomonitoring may serve several purposes: to identify unknown chemical exposures, monitor temporal trends or changes in exposure, and assess levels of exposure throughout different populations and geographical areas [8]. However, biological monitoring data relies on reference values to be able to interpret the level of exposure. There are few comparable values for non-occupational exposure [9].

**Figure 1:**
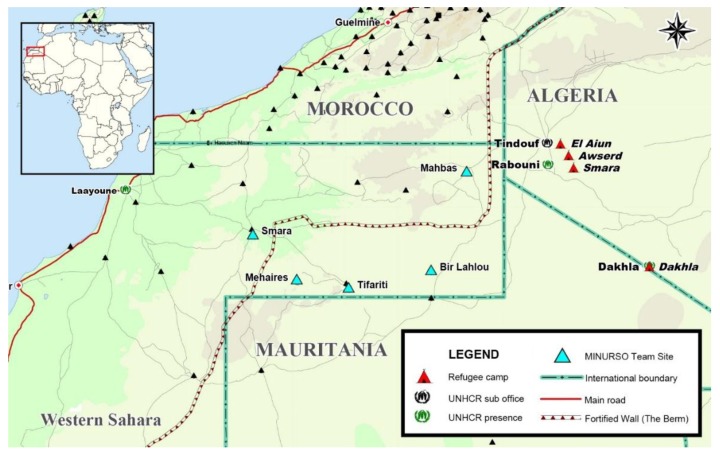
Map of Western Sahara and outline of the Saharawi camp area, showing the different camps, used with permission [22]. Water samples were collected from each of the four refugee camps marked with red triangles on the map.

In this study, we have assessed concentrations of selected elements in drinking water and urine from women and children living in the Saharawi refugee camps in the Algerian desert, where the refugees have been situated since 1975. The approximately 165,000 refugees live in four different camps—Boujador/Smara, El Aiun, Auserd, and Dakhla—located within the same area (Figure 1). The refugees live in a harsh desert environment, which makes it difficult to cultivate food crops and with limited water supply. Previous surveys have revealed poor water quality in these refugee camps, such as high levels of fluoride [10], together with high levels of iodine [11,12] and nitrate [13]. High intake of iodine and fluoride may be a health risk, as excessive iodine intake may lead to thyroid disorders [14], and excessive fluoride intake may lead to fluorosis [15]. There are no guideline values from the WHO for iodine concentration in drinking water, but several Chinese studies have found harmful effects of iodine concentrations in drinking water between 150–300 µg/L [16,17,18]. The guideline values of nitrite and nitrate in drinking water are 3 mg/L and 50 mg/L, respectively. This is due to risk of methaemoglobinaemia, where bottle-fed infants are particularly vulnerable to high intakes due to high intake of water and small body size. Thus, high exposure nitrite and nitrate from drinking water is a concern.

Reverse osmosis has been implemented for the water sources with the poorest water quality. However, the capacity of the reverse osmosis plants is not large enough to supply treated water to all camp residents [19]. In 2015, Vivar and colleagues made a thorough inspection of the water quality, including raw water, treated water, and water distributed to the hospitals. They found high levels of fluoride, sodium, sulfate, chloride, and nitrates [19]. However, the study did not include distributed public water, which is the refugees’ drinking supplies. No systematic biomonitoring has been conducted in this population, but some studies have revealed alarmingly high concentration of iodine in urine, which may lead to increased prevalence of goiter and disturbed thyroid hormone synthesis [12,20,21]. In this study, we aim to explore concentrations of selected elements in distributed drinking water from households in the Saharawi refugee camps. Moreover, we examined the urinary excretion of selected elements and compared the data with relevant literature values. We also explored whether the trace element concentration in urine relevant for thyroid hormone synthesis (iodine, vanadium, selenium, arsenic, and bromine) may be related to thyroid disturbances among the women and children.

## 2. Materials and Methods 

### 2.1. Subjects

Being part of a larger health study from 2013, the sampling selection and procedure have previously been described [21,23]. In total, 296 children born between 2010 and 2011 and 77 women (mothers of the recruited children) were included in this study. The selection was stratified on camps, and all camps were represented in the sample.

### 2.2. Water and Urine Samples

According to Vivar et al. [19] and the Saharawi Ministry of Public Health, the general drinking-water supply originates from three different sources, which includes one or several camps:

- Water source 1: El Aiun and Auserd. Water is supplied from groundwater wells and reverse osmosis plant for both of the camps. The plant does not have capacity to serve treated water continuously, hence each camp receives treated water for 21 days and raw water for 21 days in different turns. A chlorination step is required for all distributed water.
- Water source 2: Bojadour/Smara. Water is supplied from ground water wells and the reverse osmosis plant. A chlorination step is required for all distributed water.
- Water source 3: Dakhla. Water is supplied from groundwater wells followed by chlorination.

The water from the main water sources are distributed to the respective camps by trucks and kept in water storage containers from which the refugees may retrieve their water. We randomly collected samples of distributed water from each of the camps, in total 24 samples. The water samples were collected during the time span 22 April–2 July 2013. Six samples of distributed public drinking water were collected from each camp—El Aiun, Auserd, Dakhla, and Bojadour/Samara. Previous studies have reported that the water quality in Smara and Dakhla is better (raw water of better quality and purified water) than the water quality in El Aiun and Auserd (raw water with high concentration of trace elements and only partially purified water) [11,12,19]. Therefore, in the description of data, the water quality is described according to Zone 1 including Smara and Dakhla and Zone 2 including El Aiun and Auserd. Water samples were collected in 15 mL Falcon^TM^ tubes (Corning, New York, NY, USA), and stored without addition of any preserving agents at −20 °C.

During 22 April–5 June 2013 samples of spot urine from women and children were collected using the Vacuette Urine System with a transfer device (Greiner Bio-One, Krensmünster, Austria). The samples were stored at −20 °C pending analysis.

### 2.3. Chemical Analyses

In the laboratory, the deep-frozen water samples were thawed and kept dark at 4 °C in a refrigerator. Bromine, chlorine, and iodine in drinking water were quantified in an alkaline solution (5% NH_4_OH (*v/v*) by means of the Agilent 8800 Triple Quadrupole ICP-MS (Agilent, Santa Clara, CA, USA) using oxygen reaction mode. Iodine was determined on mass 127. ^129^I was used for correction of non-spectral interferences. Every other elements were determined in an acidic solution (5% nitric acid (*v/v*)) by means of the Agilent 8800 ICPMS-QQQ using either oxygen reaction mode or helium collision mode, and indium and bismuth as internal standard. The anions fluoride, chloride, nitrate, nitrite, and sulphate were determined by means the Thermo Scientific Dionex ICS-2100 ion chromatography system (Waltham, MA, USA).

The deep-frozen samples of spot urine were thawed and thoroughly homogenized at room temperature. Subsequently, aliquots of 1.00 mL of urine were diluted to 10.0 mL with an alkaline solution (BENT), containing 4% (*w/v*) 1-Butanol, 0.1% (*w/v*) H_4_EDTA, 2% (*w/v*) NH_4_OH, and 0.1% (*w/v*) Triton™, X-100, and analyzed for arsenic, bromine, chlorine, iodine, iron, lead, selenium, sulphur, vanadium, and uranium concentrations by means of the Agilent 8800 ICPMS-QQQ using oxygen reaction mode or helium collision mode and indium and bismuth as internal standard. Iodine was determined on mass 127. ^129^I was used for correction of non-spectral interferences. Reagents of analytical grade or better and deionized water (>18 MΩ) were used throughout. A conformance test between volume and weight of spot urine samples confined concentration of elements to two significant figures.

Considering water, accuracy of the analyses was checked by concurrent analysis of standard reference materials (SRM): National Institute of Standards & Technology (NIST) 1640a Trace elements in natural water and Sigma-Aldrich QC3198 Nutrient—WP (Whole volume). Accuracy in the determination of trace elements in urine was examined by analysis of Seronorm^TM^ Trace Elements Urine L-1 and Seronorm^TM^ Trace Elements Urine L-2. Regarding elements in drinking water determined using ICP-MS, except for manganese, bias was <5% for all elements with a certified value issued. Bias for manganese was 20%. Considering nitrate determined using ion-exchange chromatography (IC), the bias was also <5%. In general, trueness is considered satisfactory, however, assessment of trueness for fluoride, sulphate, bromine, and iodine in drinking water is missing due to lack of certified values for these parameters in the certified reference material analyzed.

Regarding elements in urine, the values obtained from concurrent analysis of Seronorm^TM^ L-1 and L-2 were generally within ±20% of the certified values. Limits of detection and the limits of quantification reported in Table 2 were calculated at three and 10 times the standard deviation (SD) of blank samples, respectively.

### 2.4. Thyroid Hormones and Background Characteristics

Serum samples were collected from 289 children and 76 women and analyzed for serum thyrotropin (TSH), free thyroxine (fT4), free triiodothyronine (fT3), thyroglobulin (Tg), and antibodies to thyroid peroxidase (TPOAb) and thyroglobulin (TgAb). A thorough description of sample collection and analytic procedures can be found in previously published papers [21,23] together with the reference ranges used for the different thyroid hormones and antibodies. Thyroid disturbances were assessed biochemically. Overt and subclinical hypo- and hyperthyroidism has been defined and previously described. The variable “thyroid disturbance” used in the statistical analyses includes hypo- and hyperthyroidism, as well as subjects with normal TSH and fT3 and/or fT4 outside the reference ranges for the children. None of the children had positive thyroid antibodies. Thyroid disturbances among the women included both overt- and subclinical hypo- and hyperthyroidism, women with normal TSH, fT3 and fT4 but with positive thyroid antibodies were not included. The categories for thyroid disturbances has previously been describes and published [21,23].

Background data were obtained by interview using pre-coded questionnaires. Body weight of women and children were measured to the nearest 100 g using a UNICEF digital platform scale (SECA 890, Hamburg, Germany). Height was measured to the nearest 0.1 cm using a portable UNICEF length board. Body Mass Index (BMI) was used to classify body composition in the women. The following classifications were used: underweight, normal weight, and overweight/obese, defined by a BMI < 18.5 kg/m^2^, a BMI = 18.5–24.9 kg/m^2^, and a BMI ≥ 25.0 kg/m^2^, respectively [24]. The gender-specific z-scores height-for-age (HAZ), weight-for-age (WAZ), and weight-for-height (WHZ) were used to classify children’s nutritional status [25] and were calculated using the “WHO macro” for SPSS [26]. The children were categorized as undernourished if either HAZ, WAZ, or WHZ < −2, which is also referred to as stunting, underweight, and wasting, respectively.

### 2.5. Ethical Considerations

Ethical approval was provided for the original study in 2013 (ref. 2013/192) by the Regional Committees for Medical Health Research in Norway and the Saharawi Ministry of Public Health. Supplementary approval was provided for this specific study in 2015 by the same institutions. Written informed consent was given by the mothers on behalf on themselves and the children included.

### 2.6. Statistics and Data Management

Data were analyzed using IBM SPSS version 22 (IBM Corp., Armonk, NY, USA). For values below the limit of quantification (LOQ) and limit of detection (LOD), the respective LOQ/LOD cut off divided by two was entered into the dataset. In the descriptives of the data (Table 1), the highest LOQ/LOD value was used as a cut off to assess whether the data was below LOQ/LOD. Due to low number of water samples, differences between zones were tested with Maximum Likelihood Estimation (MLE) using RStudio, when values below LOQ exceeded 15% [27]. For urine samples, the Mann Whitney U test was used.

Concentration of the different elements in urine for women and children were checked for association with Zones 1 and 2 in multiple linear regression models. For the women, the models were adjusted for BMI and age (none of the women included were lactating). For children HAZ, WAZ, WHZ, breastfeeding status, and gender were adjusted for. All dependent variables were log (2) transformed due to right skewed distribution. Residuals were checked in each model, and standard residuals above 3 or below−3 were removed from the models.

Concentrations of trace elements in urine relevant for thyroid function were explored in women and children with detected thyroid disturbances, and we tested for differences with the Mann Whitney U test.

## 3. Results

Characteristics of women and children are shown in Table 1. Median age among the women and children was 34 years and 31 months, respectively. A total of 26% of the women were normal weight, whereas 73% were overweight or obese. Among the children, 12% were underweight, 33% stunted, and 4% wasted. There were no significant differences in characteristics between Zones 1 and 2 (data not shown).

In Table 2, all LOQ values for both water and urinary analyses of elements are presented.

**Figure 2:**
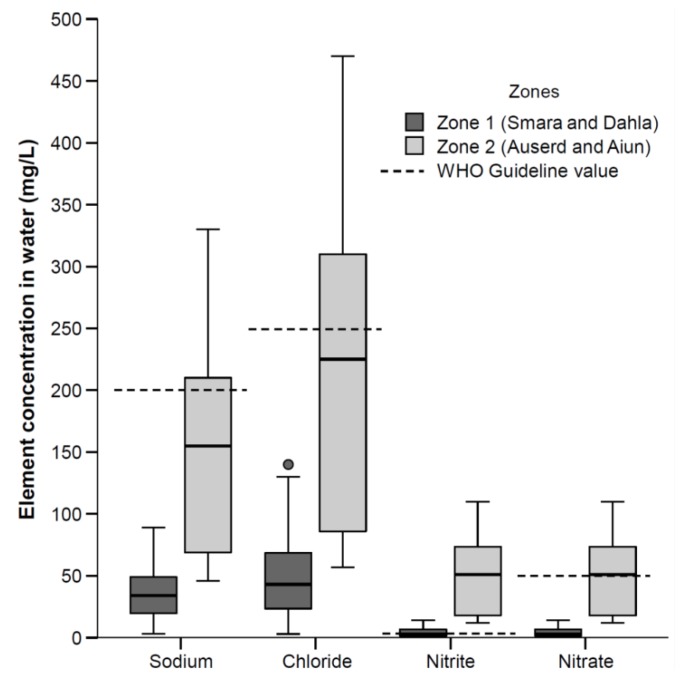
Elements in distributed public drinking water in the Saharawi refugee camps exceeding the WHO guideline values for drinking water. The dotted line the guideline value for the respective elements.

Concentration of elements and anions in distributed drinking water from Zones 1 and 2 are shown in Table 3. Regarding most of the elements in drinking water, there were significantly higher concentration in Zone 2 in comparison with Zone 1. Among the elements where guideline values are established, the 75th percentile for sodium, chloride, nitrite, and nitrate exceeded the guideline values (Figure 2). The median concentration of calcium and magnesium in Zone 1 was 45 mg/L and 3 mg/L, and 64 mg/L and 57 mg/L in Zone 2, respectively. The distributed drinking water may be classified as hard to very hard; 6.8 °dH in Zone 1 compared to 22 °dH in Zone 2.

Concentration of elements in urine among women and children are shown in Table 4 and Table 5. Among the women living in Zone 2, the median concentrations of vanadium, arsenic, bromine, selenium, and uranium exceeded the median levels of those used as reference populations [9,29]. Considering lead, women living in Zone 1 had the highest levels, which also exceeded the reference data. All urinary concentrations of elements were higher in Zone 2 than Zone 1, except for lead, which had higher median levels in women from Zone 1. Among the children, the median concentrations of vanadium, arsenic, selenium, lead, and uranium exceeded the values of the reference populations in Zones 1 and 2. Median concentrations of all elements were higher in women and children from Zone 1 than in those from Zone 2, except from iron and lead, which were higher in Zone 1.

In Table 6**,** associations between urinary concentrations of the different elements and Zones 1 and 2 are shown. For both women and children, all elements were significantly associated with zones in adjusted models, except from iron and arsenic. For the elements sulphur, chlorine, vanadium, bromine, selenium, iodine, and uranium, there was a positive and significant association with zones, where the urinary concentrations were higher in Zone 2 for all the mentioned elements except from lead, where the association was negative. Vanadium and iodine were the two elements with most variance explained by zones, 60% and 49% for vanadium and 83% and 43% for iodine among women and children, respectively.

Overall, the prevalence of thyroid disturbances was high in the population—31% in women and 14% in children. We have previously reported data regarding thyroid function in relation with iodine status among these women and children [12,21,23]. In addition to iodine, vanadium, selenium, arsenic, and bromine may also impact thyroid hormone metabolism. The urinary concentrations of vanadium and selenium were higher in women with thyroid disturbances. However, none of the differences reached statistical significance (Table 7). For arsenic and bromine, the urinary concentrations were similar in women with or without thyroid dysfunction. Among the children, the concentrations of vanadium, selenium, arsenic, and bromine were similar among both those with normal and disturbed thyroid function (Table 7).

## 4. Discussion

Previous studies have revealed poor drinking water quality in the Saharawi refugee camps. Even though water purification systems have been implemented in the camps, the concentrations of sodium, chloride, nitrite, and nitrate still exceeded the WHO guideline values. Further on, for most elements and anions, the values were significantly higher in drinking water from Zone 2 compared to Zone 1. Urinary concentrations of vanadium, arsenic, bromine, selenium, and uranium among women and children were found to be higher than reported in relevant literature studies.

### 4.1. Trace Element Concentration in Distributed Public Drinking Water

The elements and anions that exceeded WHO guidelines, were sodium, chloride, nitrite, and nitrate, in Zone 2 (see Figure 2). In line with our findings, Vivar et al. found that raw water from the same area (El Aiun and Auserd), was high in sodium, chloride, and nitrate in addition to high levels of fluoride and sulphate [19]. According to their study, treatment with reverse osmosis reduced these high concentrations within safe levels. However, due to limited capacity of the water purification systems, the population in Zone 2 is not solely provided treated water. Thus, the treated water may be mixed with raw water in the local water storage tanks. This may explain why we found high concentrations in distributed public drinking water, where the samples solely consisted of distributed public drinking water. No health-based guideline values have been established for chloride and sodium [4]. The levels of nitrite and nitrate may be of possible health concern, and the WHO guideline value is set due to risk of methaemoglobinaemia, where infants and young children are particularly vulnerable groups [4]. Nitrate may also inhibit iodide uptake into the thyroid gland [4]. Only 7% of the children under six months of age living in the refugee camps are exclusively breastfed, and a large share is given infant formula mixed with water [31]. Consequently, it cannot be ruled out that infants and young children are exposed to high levels of nitrite and nitrate.

The median vanadium concentration in Zone 2 was 55 µg/L, significantly higher than in Zone 1. No guideline value has been established by the WHO, even though toxic effects have been reported in human studies [32]. Italy has set an upper limit in drinking water at 140 µg/L [33]. However, typical values of vanadium in drinking water are below 1 µg/L, and when detected, it is rarely higher than 20 µg/L [34]. Other West-African studies have found far lower vanadium concentrations in the drinking water; an Algerian study found concentrations between 15–27 µg/L [35], and in a Ghanaian study, the concentrations were below 1 µg/L [36].

### 4.2. Associations between Zones and Urinary Excretion of Elements

In both women and children, urinary excretion of most of the elements were significantly associated with the zone they lived in (Table 6). Considering that the refugees are living under quite homogenous conditions and that they all receive the same food basket [37], this indicates that the differences in urinary concentration of these elements between zones could be attributed to drinking water. The only exceptions were arsenic and iron, for which their concentrations in drinking water were not associated with their respective urinary concentrations. Lead was significantly higher in Zone 1 compared with Zone 2.

### 4.3. Elements in Urine

Considering women, concentrations of vanadium, arsenic, bromine, selenium, and uranium in urine in Zone 2 exceeded the values of the reference studies. The urinary lead excretion in women living in Zone 1 was higher than reference values. Regarding the children, the urinary concentration of vanadium, arsenic, selenium, lead, and uranium in both Zone 1 and Zone 2 were higher than the respective reference values reported in the German reference study [30]. However, the reference studies used for both women and children were from European populations. Reference studies from refugee settings, or from low-income populations from a similar area would have been preferred. As data on bio-monitoring in such populations remain scarce, the best suited studies for the life-stage group were chosen.

### 4.4. Elements in Urine Attributable to Their Occurrence in Drinking Water

In general, considering a non-exposed population, urinary concentrations of vanadium are between 0.1–0.2 µg/L [9,29,30,38]. However, the p75-value for both women and children in Zone 2 were 3–4-fold higher. Thus, it seems evident that both the children and women in the refugee camps have a higher exposure to vanadium compared to non-occupationally exposed populations. The relatively high vanadium concentration found in drinking water, as well as association with zones, suggest that the water is a significant source of the urinary vanadium concentrations in women and children.

High iodine concentration in urine among women and children has previously been examined and published [21,23,39]. These studies found a high prevalence of biochemically assessed thyroid dysfunction among both women and children, which might have been caused by excessive iodine intakes. This has been suggested to be caused by high iodine concentration in drinking water and animal milk as a secondary source [12].

The urinary uranium concentrations for the women and children were substantially higher than what was measured in the reference studies [9,29,30]. Cross-sectional data from a non-occupational exposed population from the US NHANES study found a median urinary uranium concentration of 0.0118 µg/L with 5–95-percentile of in the range of 0.00142–0.0345 µg/L [40]. In a study from South Carolina, an area with previously known high uranium exposure from drinking water, the median urinary uranium excretion was 0.162 µg/L [41], which is comparable to that found in the Saharawi refugee camps. The WHO states that uranium intake through food is between 1–4 µg/day and that drinking water may be a substantial source in areas where uranium is present in the water [4]. Concentration of uranium in drinking water was associated with both zones, and water may be a significant source in this area.

### 4.5. Elements in Urine Elements Attributable to Other Sources

Urinary selenium usually does not exceed 30 µg/L [42], in most parts of the world except for high-selenium areas where mean values of 140 µg/L in populations without selenosis have been reported [43]. Median urinary concentrations of selenium among women in both zones were higher than usually seen. Likewise, high urinary selenium concentrations were found with respect to the children, with median values in both Zones 1 and 2 exceeding the data from the German reference study [30]. It is noted that the selenium intake in Europe is generally low. The high urinary selenium seen in our study does not seem not to originate from the drinking water alone, which was far below the WHO guideline value of 40 µg/L (Table 3). Thus, food is a likely additional source of selenium such as cereals, which are common foods among the refugees [44].

Urinary arsenic concentrations are associated with concentrations of inorganic arsenic in drinking water but may also originate from dietary sources such as seafood or rice [45]. Exposure to inorganic arsenic from drinking water is considered a major health concern by the WHO [46]. However, in the present study, the inorganic arsenic is not measured in the drinking water, nor the urinary samples. The concentration of total arsenic in the drinking water was low in Zone 1. In Zone 2, it was higher but did not exceed the WHO guideline value of 10 µg/L, which was made provisional because of practical difficulties in removing arsenic from drinking water [4]. In populations not exposed from drinking water or occupation, normal concentrations of urinary arsenic are 5–50 µg/L [47]. Accordingly, the low arsenic concentrations in the drinking water, and higher urinary concentrations indicates that the source probably is dietary. As rice and seafood are major sources of dietary arsenic, and since the refugees consume very little fish, the source is likely to be rice [44].

Urinary lead excretion was higher than reported in reference studies. Interestingly, the urinary lead excretion was highest in Zone 1, in contrast to the other measured elements. For children, the urinary lead concentration was approximately three times higher than in the reference study, in both zones, and for the women in Zone 1, it was approximately five times higher than the reference studies [9,29,30]. In comparison, a British study from a non-occupational exposed adult population found a median urinary concentration of 12 µg/L [48]. Although the urinary lead excretion is not alarmingly high, even low exposures to lead may increase risk of negative health effects [49] and should therefore be reduced to a minimum. There were little differences in lead concentrations in water between Zones 1 and 2, and no association between urinary concentrations and zones. Thus, the lead exposure as measured in urine may be attributable to sources other than water.

### 4.6. Trace Elements in Urine Relevant for Thyroid Metabolism

Iodine is essential for thyroid hormone metabolism, and the relation between thyroid function and iodine status from these data has previously been examined. No relation between UIC and thyroid dysfunction were found among women and children [12,21,23]. However, UIC from spot urine samples is a poor indicator of individual iodine status. Further, both women and children from Zones 1 and 2 had UIC levels above the WHO cut-off indicating excessive iodine intakes [50], hence the variation in exposure was small. A study in the same population has revealed an association between breast milk iodine and thyroid dysfunction [12]. Since breast milk iodine concentration serves as a good indicator of iodine status among lactating women, we would argue that the high iodine exposure may be a plausible cause of the high prevalence of thyroid dysfunction found among the Saharawi women and children. Differences in urinary concentration of other trace elements relevant for thyroid hormone metabolism (vanadium, selenium, arsenic, and bromine) were explored. There were no significant differences in either of the selected trace elements in urine between women and children with normal and disturbed thyroid function. However, concentrations in urine may not serve as a suitable biomarker, and in addition, only one spot urine sample per participant was collected. Thus, excessive exposure of trace elements and possible association with thyroid function could be further explored in this population, as the prevalence of thyroid hormone disturbances was very high.

## 5. Conclusions

Previous studies have found poor drinking water quality including high concentrations of some trace elements in the Saharawi refugee camps. In recent years, an effort has been made to improve the water quality using purification by reverse osmosis. In the areas with purified water and raw water of better quality (Zone 1), the concentrations of elements seemed to be within acceptable levels. However, in the areas with partially purified water (Zone 2), some elements exceeded the WHO guideline values. Additionally, urinary concentration of selected elements was higher in Zone 2 than Zone 1 and exceeded values from reference studies. Further effort should be made to improve the water quality by increasing the share of purified water distributed in order to ensure the population access to clean and safe drinking water, fulfilling their right to safe drinking water.

## Figures and Tables

**Table 1:** Background characteristics of Saharawi women and children ^a^.

Characteristics Women	Zone 1 (*n* = 34)	Zone 2 (*n* = 43)	Total (*n* = 77)
Age, years ^b^	33.0 (25.3–36.8)	38.0 (30.8–40.0)	34.0 (30.0–39.0)
Height, cm	158.1 ± 5.6	156.0 ± 6.1	156.9 ± 6.9
Weight, kg	70.0 ± 11.3	70.4 ± 14.0	70.2 ± 12.8
BMI, kg/m^2^	27.1 ± 5.2	27.9 ± 5.3	28.5 ± 5.1
<18.5	0	1 [2.3]	1 [1.3]
18.5–24.9	9 [26.5]	11 [25.6]	20 [26.0]
≥ 25	25 [73.6]	31 [72.2]	56 [72.7]
Household size, number	5.5 ±1.8	5.3 ± 1.7	5.4 ± 1.7
Children < 5 years	1.5 ± 0.7	1.4 ± 0.5	1.4 ± 0.6
**Characteristics Children**	**Zone 1 (*n* = 192)**	**Zone 2 (*n* = 104)**	**Total (*n* = 296)**
Age, months	30.3 (25.1–34.8)	32.7 (24.9–35.4)	31.4 (25.2–35.2)
Male	83 [43.2]	58 [55.8]	141 [47.6]
Female	109 [56.8]	46 [44.2]	155 [52.4]
Still breastfed, yes	23 [12.0]	18 [17.3]	41 [13.9]
Weight-for-age, z-score ^c^	−1.0 ± 0.9	−1.0 ± 0.9	−1.0 ± 0.9
<−2 (underweight)	20 [10.4]	14 [13.5]	34 [11.5]
Length/height-for-age, z-score ^c^	−1.6 ± 1.1	−1.6 ± 1.0	−1.6 ± 1.0
<−2 (stunted)	61 [31.9]	37 [35.6]	98 [33.2]
Weight-for-length/height, z-score ^c^	−0.1 ± 1.0	−0.2 ± 0.9	−0.2 ± 1.0
<−2 (wasted)	7 [3.7]	4 [3.8]	11 [3.7]

**^a^** Values are presented as mean ± SD, median (P_25_–P_75_), and n [%]. ^b^ 3 missing from age women. ^c^ 1 missing from HAZ and WHZ.

**Table 2:** Limit of quantification (LOQ) for all elements and anions in drinking water and urine. Percentage of samples with unquantifiable (<LOQ) and undetectable (<LOD) element concentrations in water, and in urine among women and children, respectively.

Element	Water Samples (*n* = 24)			Urine Samples				
					Women (*n* = 80)		Children (*n* = 296)	
	LOQ (µg/L)	<LOQ (%)	<LOD (%)	LOQ (µg/L)	<LOQ (%)	<LOD (%)	<LOQ (%)	<LOD (%)
^a, c^ Chlorine (Cl)	38	0	0	2.8 × 10^3^	0	0	0	0
^a^ Potassium (K)	19	4	0	nd				
^a^ Sulphur (S)	nd			2.0 × 10^3^	0	0	0	0
^a^ Aluminum (Al)	5.7	8	0	nd				
^a^ Arsenic (As)	28 × 10^−3^	0	0	6.3	1		3	0
^a^ Barium (Ba)	5.2	13	4	nd				
^a^ Boron (B)	16	0	0	nd				
^a^ Bromine (Br)	92 × 10^−3^	0	0	42	0	0	0	0
^a^ Cadmium (Cd)	18 × 10^−3^	92	67	nd				
^a^ Calcium (Ca)	28	0	0	nd				
^a^ Cerium (Ce)	7.4 × 10^−3^	46	8	nd				
^a^ Cesium (Cs)	38 × 10^−3^	75	54	nd				
^a^ Chromium (Cr)	0.54	88	38	nd				
^a^ Copper (Cu)	0.56	50	13	nd				
^a^ Iodine (I)	0.18	0	0	6.3	0	0	0	0
^a^ Iron (Fe)	2.3	33	0	15.0	9		6	0.3
^a^ Lead (Pb)	0.12	71	38	5.6	49	34	13	7
^a^ Lithium (Li)	2.7	13	8	nd				
^a^ Magnesium (Mg)	8.1	0	0	nd				
^a^ Manganese (Mn)	0.25	29	8	nd				
^a^ Molybdenum (Mo)	23 × 10^−3^	0	0	nd				
^a^ Nickel (Ni)	0.84	92	38	nd				
^a^ Rubidium (Rb)	0.19	13	8	nd				
^a^ Selenium (Se)	0.27	17	8	0.79	0	0	0	0
^a^ Sodium (Na)	13 × 10^1^	0	0	nd				
^a^ Silicon (Si)	6.8	0	0	nd				
^a^ Strontium (Sr)	0.19	0	0	nd				
^a^ Thallium (Tl)	6.2 × 10^−3^	58	29	nd				
^a^ Vanadium (V)	25 ×10^−3^	0	0	80 × 10^−3^	0	0	0	0
^a^ Zinc (Zn)	3.2	29	13	nd				
^a^ Uranium (U)	4.5 × 10^−3^	0	0	0.13 ^d^	25	0	23	0
^b^ Fluoride (F^−^)	0.14 × 10^3^	25	21					
^b^ Chloride (Cl^−^)	0.49 × 10^3^	0	0					
^b^ Nitrate (NO_3_^−^)	0.35 × 10^3^	0	0					
^b^ Nitrite (NO_2_^−^)	0.27 × 10^3^	0	0					
^b^ Sulfate (SO_4_^2−^)	0.34 × 10^3^	0	0					

nd: not determined. ^a^ Agilent 8800 ICP-QQQ; ^b^ Dionex ICS-2100; ^c^ NH4OH used in the alkaline sample preparation contain chlorine; nevertheless, due to the high level of chlorine in urine, the significantly higher LOQ compared to the acidic sample preparation have no influence on the obtained data; ^d^ In one batch the LOQ was 0.13 µg/L, mean LOQ was 0.08 µg/L.

**Table 3:** Concentration of elements and anions in distributed drinking water from zones 1 and 2 in the Sahara refugee camps and comparison with guideline values from WHO or USEPA.

Elements and Anions	Zone 1 (*n* = 12)		Zone 2 (*n* = 12)		*p*	Total (*n* = 24)		WHO/US Guideline Values [4,28]
	Median	P_25_–P_75_	Median	P_25_–P_75_		Median	P_25_–P_75_	
V, µg/L	0.93	0.28–1.9	55	47–62	<0.001	20	0.85–73	-
Fe, µg/L	3.9	2.8–5.5	1.1	1.1–4.2	0.039	3.0	1.1–5.1	2000
As, µg/L	0.12	0.086–0.21	2.8	1.9–4.5	<0.001	0.83	0.12–2.9	10
Se, µg/L	0.50	0.14–1.3	5.0	2.0–7.0	<0.001 ^a^	1.6	0.42–5.3	40
I, µg/L	80	73–92	250	230–270	<0.001	100	80–260	-
Pb, µg/L	<0.12	<0.12–0.20	<0.12	<0.12 ≤ 0.12	0.689	<0.12	<0.12–0.13	10
U, µg/L	0.7	0.04–2.1	5.1	3.9–7.7	<0.001	2.9	0.44–5.2	30
Li, µg/L	11	2.5–27	59	22–80	<0.001 ^a^	23	9.5–60	-
B, µg/L	99	73–270	330	230–430	0.002	230	93–400	2400
Na, mg/L	34	15–53	155	68–210	<0.001	62	34–220	200
Mg, mg/L	3.0	1.2–5.3	57	22–78	<0.001	11	2.9–58	-
Al, µg/L	8.3	6.9–11	8.7	7.7–13	0.583 ^a^	8.6	7.6–11	200 ^b^
Si, mg/L	4.2	0.78–7.7	21	20–21	<0.001	13	2.7–21	-
K, µg/L	1300	580–2200	6200	2600–8900	0.002 ^a^	2500	1300–6400	-
Ca, mg/L	45	5.0–95	64	55–66	0.977	64	21–76	-
Cr, µg/L	<0.54	<0.54 ≤ 0.54	<0.54	<0.54–0.6	0.999 ^a^	<0.54	<0.54 ≤ 0.54	50
Mn, µg/L	0.6	0.5–1.3	<0.25	<0.25–0.7	0.097 ^a^	0.5	<0.25–1.0	400
Ni, µg/L	<0.84	<0.84 ≤ 0.84	<0.84	<0.84 ≤ 0.84	0.999 ^a^	<0.84	<0.84 ≤ 0.84	70
Cu, µg/L	<0.56	<0.56–0.89	<0.56	<0.56–0.95	0.999 ^a^	<0.56	<0.56–0.89	2000
Zn, µg/L	6.0	<3.2–590	5.9	<3.2–35	0.225 ^a^	5.9	<3.2–160	3000
Rb, µg/L	1.0	0.16–1.8	3.1	1.4–4.3	<0.001 ^a^	1.5	0.6–3.1	-
Sr, µg/L	550	62–1200	2100	1900–2600	<0.001	1600	390–2200	17,000 ^b^
Mo, µg/L	0.37	0.08–0.78	2.8	1.4–3.9	<0.001	1.1	0.30–2.9	70
Cd, µg/L	<0.02	<0.02 ≤ 0.02	0.02	<0.02 ≤ 0.02	0.941 ^a^	<0.02	<0.02 ≤ 0.02	3
Cs, µg/L	0.09	<0.038–0.4	<0.038	<0.038 ≤ 0.038	0.999 ^a^	<0.038	<0.038–0.14	-
Ba, µg/L	20	3.7–25	11	9.3–14	0.478 ^a^	12	8.0–20	700
Ce, µg/L	0.01	<0.007–0.02	<0.007	<0.007–0.02	<0.001 ^a^	0.008	<0.007–0.02	-
Tl, µg/L	0.02	<0.006–0.05	<0.006	<0.006–0.006	<0.001 ^a^	<0.006	<0.006–0.02	2 ^b^
F^−^, mg/L	0.17	0.02–0.32	1.1	0.8–1.2	<0.001 ^a^	0.56	0.12–1.1	1.5
Cl^−^, mg/L	43	18–73	230	84–310	<0.001	80	43–240	250
NO_2_^−^, mg/L	2.2	1.1–2.8	5.4	2.8–5.9	<0.001	2.8	2.1–5.5	3
NO_3_^−^, mg/L	2.7	1.4–8.3	51	18–74	<0.001	13	2.6–54	50
SO_4_^2−^, mg/L	88	7.0–200	270	170–360	0.004	200	50–290	500

Differences tested with Mann Whitney U test, values marked with ^a^ Tested with MLE. ^b^ Guideline values detained from US Environmental protection Agency.

**Table 4:** Concentration of elements in urine among Saharawi women living in Zones 1 and 2 in comparison with other published data.

Elements and Anions	Unit	Zone 1 (*n* = 34)		Zone 2 (*n* = 43)		Total (*n* = 77)		Reference Data			
								Hoet et al., 2013 [9]		Morton et al., 2014 [29]	
		Median	P_25_–P_75_	Median	P_25_–P_75_	Median	P_25_–P_75_	Median	URL ^a^	Median	P_95_
Sulphur	µg/L	620	330–910	1000	540–1600	820	485–1200	-	-	-	-
Chlorine	mg/L	2800	1200–4800	3600	2000–5400	3100	1800–5050	-	-	-	-
Vanadium	µg/L	0.19	0.11–0.37	2.6	1.3–5.8	0.83	0.22–3.1	0.25	1.5	1.6	3.8
Iron ^b^	µg/L	22	9.0–36	22	12–32	22	11–33	-	-	-	-
Arsenic	µg/L	28	15–48	31	18–53.	31	16–51	14.1	300	10.5	152.4
Bromine	mg/L	5.0	3.6–7.5	6.9	5.2–9.5	6.1	4.8–8.6	-	-	2.3	5.4
Selenium	µg/L	36	25–54	58	31–88	46	28–64	25.1	80	13.4	33.4
Iodine	µg/L	150	92–230	800	630–1500	500	170–900	-	-	-	-
Lead	µg/L	2.6	1.2–3.9	0.05	0.05–1.1	1.1	0.05–2.9	0.87	4	0.5	7.6
Uranium	µg/L	<0.13	<0.13 ≤ 0.13	0.22	0.15–0.32	0.15	<0.13–0.26	<0.007	0.05	-	-

^a^ Upper Reference level, established by Hoet et al. [9]. ^b^ 49% < LOQ for iron, thus the values should be interpreted with caution.

**Table 5:** Concentration of elements in urine among Saharawi children from Zones 1 and 2 in comparison with other published data.

Elements and Anions	Unit	Zone 1 (*n* = 192)		Zone 2 (*n* = 104)		Total (*n* = 296)		Reference Data	
								Heitland et al., 2005 [30]	
		Median	P_25_–P_75_	Median	P_25_–P_75_	Median	P_25_–P_75_	Median	Min–Max
Sulphur	µg/L	830	480–1200	1300	620–1900	920	520–1400	-	-
Chlorine	mg/L	2700	1600–4800	4500	1850–6200	3300	1700–5300	-	-
Vanadium	µg/L	0.44	0.26–0.77	4.4	1.9–7.8	0.73	0.33–3.0	<0.056 ^a^	<0.056 ^a^–0.16
Iron	µg/L	28	18–52	27	16–42	28	17–46		
Arsenic	µg/L	27	12–68	36	22–68	32	16–68	25	1-260
Bromine	mg/L	6.1	4.5–8.7	7.8	5.0–11	6.5	4.6–9.4	-	-
Selenium	µg/L	43	25–65	72	32–100	50	27–76	17	4-39
Iodine	µg/L	320	180–480	1400	680–2500	430	240–1000	-	-
Lead	µg/L	4.1	2.6–6.8	2.8	1.1–6.3	3.7	2.1–6.7	1.3	0.1-4.6
Uranium	µg/L	0.14	<0.13–0.20	0.27	0.18–0.47	0.18	<0.13–0.27	0.004	<0.004 ^a^–0.003

^a^ Reported LOQ value from Heitland et al. [30].

**Table 6:** Concentration of different chemical elements in urine among women and children in association with zones.

Elements in Urine Women	Adjusted Coefficient (95% CI) ^a^	*p*	Stand Beta	*R* ^2^
Sulphur (*n* = 74)	0.8 (0.4, 1.3)	<0.001	0.225	0.20
Chlorine (*n* = 74)	0.5 (0.04, 1.0)	0.033	0.244	0.12
Vanadium (*n* = 74)	4.0 (3.2, 4.8)	<0.001	0.768	0.60
Iron (*n* = 74)	0.2 (−0.5, 0.8)	0.644	0.055	0.03
Arsenic (*n* = 74)	0.3 (−0.3, 0.9)	0.333	0.116	0.02
Bromine (*n* = 74)	0.6 (0.2, 0.9)	<0.001	0.397	0.19
Selenium (*n* = 74)	0.7 (0.3, 1.1)	0.001	0.370	0.20
Iodine (*n* = 66)	3.0 (2.6, 3.3)	<0.001	0.867	0.83
Lead (*n* = 74)	−3.6 (−4.5, −2.6)	<0.001	−642	0.42
Uranium (*n* = 72)	1.2 (0.8, 1.6)	<0.001	0.581	0.35
**Elements in Urine Children**				
Sulphur (*n* = 293)	0.4 (6.3, 8.4)	0.001	0.194	0.15
Chlorine (*n* = 287)	0.5 (0.2, 0.7)	<0.001	0.220	0.15
Vanadium (*n* = 294)	3.0 (2.6, 3.4)	<0.001	0.183	0.49
Iron (*n* = 288)	−0.1 (−0.4, 0.2	0.449	0.161	0.07
Arsenic (*n* = 292)	0.2 (−0.2, 0.6)	0.314	0.059	0.06
Bromine (*n* = 293)	0.3 (0.1, 0.4)	0.002	0.078	0.08
Selenium (*n* = 292)	0.5 (0.3, 0.8)	<0.001	0.221	0.16
Iodine (*n* = 294)	2.1 (1.8, 2.4)	<0.001	0.617	0.43
Lead (*n* = 294)	−1.4 (−1.9, −1.0)	<0.001	−0.337	0.13
Uranium (*n* = 290)	1.1 (0.9. 1.3)	<0.001	0.495	0.25

All dependent variables are log (2) transformed. Categories for zones: 0 = zone 1, 1 = zone 2. ^a^ Children: adjusted for HAZ, WAZ, WHZ, age, gender, and breastfeeding status. Categories for gender child: 0 = male, 1 = female). Categories for HAZ, WAZ and WHZ: 0 = normal nutrition status, 1 = undernourished. Categories for breastfeeding status: 0 = no, 1 = yes). Women: adjusted for BMI and age.

**Table 7:** Concentration of selected trace elements in urine among women and children with thyroid disturbances ^a^.

	Thyroid Disturbances Women ^b^			Thyroid Disturbances Children ^c^		
Trace Elements	Yes (*n* = 20)	No (*n* = 44)	*p*	Yes (*n* = 40)	No (*n* = 246)	*p*
Iodine, µg/L	690 (283–998)	390 (173–798)	0.202	365 (173–685)	445 (258–1100)	0.151
Vanadium, µg/L	1.5 (0.3–6.0)	0.8 (0.3–2.5)	0.171	0.5 (0.3–2.4)	0.8 (0.4–3.2)	0.123
Selenium, µg/L	49 (29–69)	41 (27–65)	0.805	44 (21–77)	52 (28–76)	0.301
Arsenic, µg/L	29 (17–34)	32 (15–49)	0.385	25 (12–57)	33 (17–74)	0.118
Bromine, mg/L	6.1 (5.0–9.2)	6.2 (4.8–8.1)	0.778	6.0 (4.7–9.2)	6.6 (4.6–9.5)	0.667

^a^ Values are presented as median (p25–p75). Differences between groups are tested by Mann Whitney U. Categorisation and results regarding thyroid disturbances have previously been described and published, as well as iodine status [21,23]. ^b^ Pregnant women not included. ^c^ 10 children missing from blood samples.
